# Statistical Improvement of rGILCC 1 and rPOXA 1B Laccases Activity Assay Conditions Supported by Molecular Dynamics

**DOI:** 10.3390/molecules28217263

**Published:** 2023-10-25

**Authors:** María P. C. Mora-Gamboa, María C. Ferrucho-Calle, Leidy D. Ardila-Leal, Lina M. Rojas-Ojeda, Johan F. Galindo, Raúl A. Poutou-Piñales, Aura M. Pedroza-Rodríguez, Balkys E. Quevedo-Hidalgo

**Affiliations:** 1 Laboratorio de Biotecnología Molecular, Grupo de Biotecnología Ambiental e Industrial (GBAI), Departamento de Microbiología, Facultad de Ciencias, Pontificia Universidad Javeriana, Bogotá 110231, Colombiamaria.ferrucho@javeriana.edu.co (M.C.F.-C.); ldardilal@ufpso.edu.co (L.D.A.-L.); 2 Laboratorio de Biotecnología Vegetal, Grupo de Investigación en Asuntos Ambientales y Desarrollo Sostenible (MINDALA), Departamento de Ciencias Agrarias y del Ambiente, Universidad Francisco de Paula Santander, Ocaña 546552, Colombia; 3 Departamento de Química, Universidad Nacional de Colombia, Bogotá 111321, Colombia; 4 Laboratorio de Microbiología Ambiental y Suelos, Grupo de Biotecnología Ambiental e Industrial (GBAI), Departamento de Microbiología, Facultad de Ciencias, Pontificia Universidad Javeriana, Bogotá 110231, Colombia; 5 Laboratorio de Biotecnología Aplicada, Grupo de Biotecnología Ambiental e Industrial (GBAI), Departamento de Microbiología, Facultad de Ciencias, Pontificia Universidad Javeriana, Bogotá 110231, Colombia; bquevedo@javeriana.edu.co

**Keywords:** ABTS, acetate and citrate buffer, laccases, molecular docking and dynamics

## Abstract

Laccases (E.C. 1.10.3.2) are glycoproteins widely distributed in nature. Their structural conformation includes three copper sites in their catalytic center, which are responsible for facilitating substrate oxidation, leading to the generation of H_2_O instead of H_2_O_2_. The measurement of laccase activity (UL^−1^) results may vary depending on the type of laccase, buffer, redox mediators, and substrates employed. The aim was to select the best conditions for rGILCC 1 and rPOXA 1B laccases activity assay. After sequential statistical assays, the molecular dynamics proved to support this process, and we aimed to accumulate valuable insights into the potential application of these enzymes for the degradation of novel substrates with negative environmental implications. Citrate buffer treatment T2 (CB T2) (pH 3.0 ± 0.2; λ_420nm_, 2 mM ABTS) had the most favorable results, with 7.315 ± 0.131 UL^−1^ for rGILCC 1 and 5291.665 ± 45.83 UL^−1^ for rPOXA 1B. The use of citrate buffer increased the enzyme affinity for ABTS since lower *K_m_* values occurred for both enzymes (1.49 × 10^−2^ mM for rGILCC 1 and 3.72 × 10^−2^ mM for rPOXA 1B) compared to those obtained in acetate buffer (5.36 × 10^−2^ mM for rGILCC 1 and 1.72 mM for rPOXA 1B). The molecular dynamics of GILCC 1–ABTS and POXA 1B–ABTS showed stable behavior, with root mean square deviation (RMSD) values not exceeding 2.0 Å. Enzyme activities (rGILCC 1 and rPOXA 1B) and 3D model–ABTS interactions (GILCC 1–ABTS and POXA 1B–ABTS) were under the strong influence of pH, wavelength, ions, and ABTS concentration, supported by computational studies identifying the stabilizing residues and interactions. Integration of the experimental and computational approaches yielded a comprehensive understanding of enzyme–substrate interactions, offering potential applications in environmental substrate treatments.

## 1. Introduction

Laccases (E.C. 1.10.3.2), also known as p-diphenol: dioxide oxidoreductases, are multi-copper oxidase glycoproteins widely distributed in plants, fungi, bacteria, algae, and insects [1,2,3,4]. Wood white rot fungi are extensively studied for laccase production [4,5] due to their high redox potential [6,7]. Laccases exhibit biotechnological potential, as they can oxidize various substrates, including phenolic and inorganic compounds. They have been reported in degradation processes [3], synthetic dye treatment [8,9], degradation of pesticides [10], antibiotics [11], plastic, and polyvinyl chloride [12], and the oxidation of phenolic compounds [13] underscores their versatility. The efficacy of laccases in substrate–product bioconversion has positioned them as a promising industrial biotechnological solution to mitigate waste accumulation across diverse industries [14].

**Figure 1:**
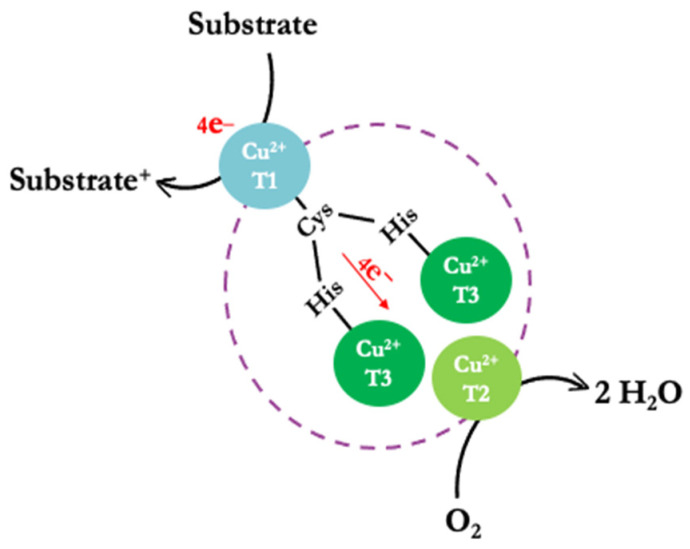
Simplified schematic representation of the reaction mechanism (substrate oxidation) of the laccases.

The structural conformation of laccases includes three types of copper ions distributed across two sites, forming a triangular matrix based on their spectroscopic properties [15,16]. The active center site consists of a mononuclear type 1 copper (CuT1) and a trinuclear copper site (TNC) that includes one type-2 copper (CuT2) and two type-3 coppers (CuT3a and CuT3b) (Figure 1) [4,17,18,19]. The CuT1 and TNC sites are at an approximate distance of 13 Å and linked through a triad of amino acids (His-Cys-His) [16].

In the active center of the enzyme, the oxidation of substrates generates H_2_O rather than H_2_O_2_ [20]. This substrate oxidation mechanism involves three steps, starting with the enzyme in its oxidized Cu (II) form at rest. CuT1 is reduced by the substrate, enabling an electron transfer from CuT1 to the trinuclear copper center (CuT3a and CuT3b) via the Cys-His pathway. Subsequently, an O_2_ molecule binds to TNC, generating an asymmetric activation; it is postulated that the O_2_-binding site restricts the access of oxidizing agents other than O_2_. Remarkably, during catalysis, no H_2_O_2_ is detected outside the laccase, indicating that the process involves a four-electron reduction of O_2_ to H_2_O (Figure 1) [4].

Spectrophotometry is a widely utilized method for detecting laccase activity due to its simplicity and sensitivity. Several common substrates are employed in activity determination techniques, such as ABTS, syringaldazine, and guaiacol [21]. Among these, ABTS has been the most frequent substrate. ABTS, in its reduced form (first oxidation state), displays a faint green color, which transitions to a dark-green color when oxidized; by measuring the intensity of this color change at a specific time, laccase detection and activity quantification occurs [22].

Enzyme assays have various applications serving to determine the presence or absence of the enzyme in a sample (qualitative determination), quantify enzyme activity (quantitative determination), demonstrate functional identity (in electrophoresis), determine specific enzyme activity (in immunoassays), and determine enzyme kinetics (to determine *V_max_* and *K_m_*). The enzyme activity is highly dependent on defined conditions such as temperature, pH, substrate type, ion nature, and strength as well as the concentrations of assay components [23]. 

The specificity of an enzyme for its substrate is influenced by several factors, including the substrate binding pocket, specific amino acid residues within the binding site, and the difference in redox potential between the active site and the substrate [24]. In the case of laccases, substrate specificity and affinity are also influenced by the pH [22]. The pH plays a crucial role in the catalytic process, which involves electron and proton transfers to or from the active site. The transfer of protons or electrons is affected by the reorganization energy associated with the reactive pH, resulting in different interactions with the substrates [25].

The interactions between an enzyme and a ligand involve complex physicochemical mechanisms that are crucial for molecular recognition between both entities. The understanding of this association relies on studying the protein–ligand binding kinetics, with a particular focus on the binding rate [26]. In recent years, computational methods have emerged as indispensable tools in comprehending various aspects of enzyme–ligand interactions. 

Computational approaches have played a vital role in elucidating catalytic mechanisms, exploring conformational changes in enzymes, deciphering interactions during the formation of enzyme–ligand complexes, and the enzyme affinity for substrates with promising applications in biotechnology, chemistry, and the environment. 

Several computational methods, varying in cost and accuracy, have played a crucial role in understanding molecular-level processes. One such method is molecular docking, which predicts the structure of the ligand–receptor complexes, providing a foundation for subsequent molecular dynamics simulations [27,28]. Molecular dynamics simulations allow the analysis of ligand–substrate interaction by assessing the stability during complex formation [29]. This approach proves beneficial in reducing the experimental costs by predicting the likelihood of substrate degradation or transformation by the enzyme under investigation. 

In this study, the statistical analysis allowed the selection of the optimal conditions for the activity assay of rGILCC 1 and rPOXA 1B laccases. The research was further supported by molecular dynamics simulations, aiming to gain valuable insights into the potential application of these enzymes in the degradation or transformation of environmentally impactful substrates.

## 2. Results

### 2.1. Optimal Experimental Designs (OED)

Table 1 shows the results of the optimal experimental designs (OED) using the rGILCC 1 and rPOXA 1B, based on the enzyme activity measurements with acetate buffer and citrate buffer. The models for both enzymes and buffers were significant (*p* < 0.0001), while the lack of fit was not significant. The analysis of factors in acetate and citrate buffer in both enzymes (rGILCC 1 and rPOXA 1B) also showed significance (*p* < 0.05). However, interactions between certain factors were not significant; for rGILCC 1 in both buffers, BC and ABC factors were not significant, and for rPOXA 1B, the interactions between ABC in the acetate buffer and BC in the citrate buffer were not significant. Additionally, the effects and contribution percentages in acetate buffer showed a negative impact of pH and wavelength factors, while the ABTS had a positive influence (statistical effect) on enzyme activity for both enzymes. Notably, the pH factor had the highest contribution percentages, accounting for 46.11 and 53.62% of the variation in enzyme activity for rGILCC 1 and rPOXA 1B, respectively.

Table 2 shows the comparison between the observed and model-predicted results of the response variable (enzyme activity, UL^−1^) for the different treatments at optimal experimental designs (OED) and at low-pH assay. At OED, the highest enzyme activities for rGILCC 1 were observed in treatments with ABG T2 and CBG T2, obtaining values of 6.30 ± 0.17 and 7.31 ± 0.13 UL^−1^, respectively. Similarly, for rPOXA 1B, treatments with ABP T2 and CBP T2 showed the highest enzyme activities, presenting values of 4773.66 ± 95.15 and 5291.67 ± 45.83 UL^−1^, respectively. Among the treatments, the T2 citrate buffer treatment (pH 3.0; wavelength 420 nm; ABTS 2 mM) yielded the highest enzyme activities for both enzymes (rGILCC 1 and rPOXA 1B). On the other hand, the lowest enzyme activity occurred in treatments ABG T7 for rGILCC 1 and CBP T11 for rPOXA 1B.

### 2.2. Low-pH Assay for rGILCC 1 and rPOXA 1B in Acetate and Citrate-Phosphate Buffers

The results of enzyme activity analysis for rGILCC 1 and rPOXA 1B at pH levels lower than those evaluated in the optimal experimental designs are in Table 2. Using acetate buffer, the enzyme activities for rGILCC 1 and rPOXA 1B were 6.67 ± 0.19 and 4819.44 ± 55.56 UL^−1^, respectively. The laccase activity in acetate buffer was slightly lower than that observed in citrate-phosphate buffer (C-PB). However, it is noteworthy that even with the use of citrate-phosphate buffer, the enzyme activities values did not exceed the highest activity levels obtained with the best treatment in citrate buffer, which were 7.037 ± 0.32 and 4884.26 ± 48.78 UL^−1^ for rGILCC 1 and rPOXA 1B, respectively (Table 2).

### 2.3. Mean Comparison

To select treatments with the highest enzyme activity (UL^−1^) a means comparison of the treatments included in both experimental designs was carried out (Table 2). The analysis shows for rGILCC 1 that treatments C-PBG OFED, ABG OFED, CBG T1, and CBG T2 resulted grouped in subset a, and in rPOXA 1B, CPB T2 treatment was the only one classified in subset a, demonstrating significant differences with the other treatments.

The citrate buffer generated the highest enzyme activities. In the subset at the top for rGILCC 1 the CBG T2 treatment (pH 3.0 ± 0.2; λ_420nm_; 2.0 mM ABTS) and at the bottom for rPOXA 1B the CBP T2 treatment (pH 3.0 ± 0.2; λ_420nm_; 2.0 mM ABTS) generated the best conditions. For both enzymes, the conditions coincided with the detection of laccase activity. All treatments that included acetate buffer generated lower enzyme activity than citrate buffer no matter the conditions used. However, the treatments with the highest enzyme activity in acetate buffer for rGILCC 1 and rPOXA 1B were ABG T2 (pH 4.0 ± 0.2; λ_420nm_; 2.0 mM ABTS) and ABP T2 (pH 4.0 ± 0.2; λ_420nm_; 2.0 mM ABTS), respectively.

In Table 2, for rGILCC 1, the ABG OFED treatment stands out, while for POXA 1B, the stand-out treatments are C-PBP OFED, ABP T2, and CBP T1. Among all the measurements, the ABG T7 treatment (pH 4.5 ± 0.2; λ_436nm_; 0.5 mM ABTS) in rGILCC 1 and CBP T11 (pH 4.5 ± 0.2; λ_436nm_; 0.5 mM ABTS) in rPOXA 1B showed the lowest enzyme activities.

### 2.4. Assay for rGILCC 1 and rPOXA 1B in Britton–Robinson Buffer at pH 3.0 and 4.0 ± 0.2

**Figure 2:**
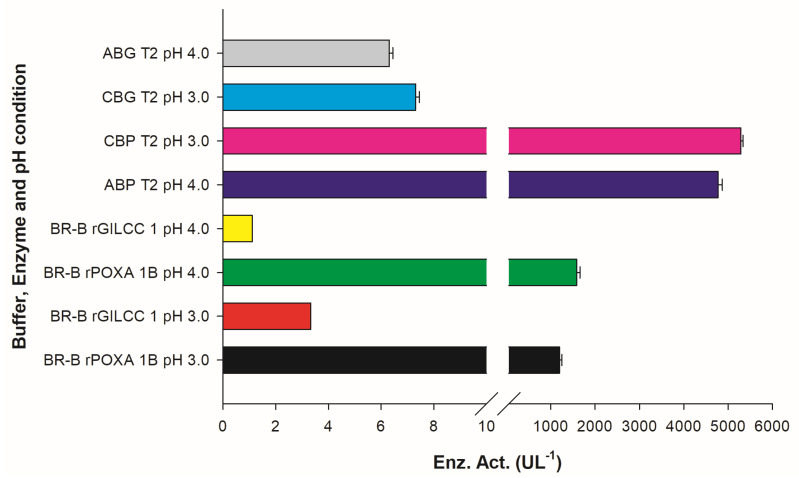
Results of enzyme activity assay for rGILCC 1 and rPOXA 1B in Britton–Robinson buffer at pH 3.0 and 4.0 ± 0.2. The best results are shown for rGILCC 1 (CBG T2, ABG T2) and rPOXA 1B (CBP T2, ABP T2) at pH 3 and 4 ± 0.2 compared to treatments at the same pH but in Britton–Robinson buffer and containing no citrate, demonstrating that the presence of the ion has a positive impact on enzyme activity.

In the Britton–Robinson buffer at pH 3 ± 0.2, the laccases activities were 3.30 ± 0.00 and 1194.44 ± 55.56 UL^−1^ for rGILCC 1 and rPOXA 1B, respectively. At pH 4.0 ± 0.2, the laccase activities were 1.11 ± 0.00 and 1583.33 ± 73.49 UL^−1^ for rGILCC 1 and rPOXA 1B, respectively. Figure 2 shows that the Britton–Robinson buffer generated better enzyme activity at pH 4.0 ± 0.2 than at pH 3.0 ± 0.2, showing the buffer’s influence and highlighting the best pH (pH 4.0 ± 0.2); even so, the enzyme activity resulted as underestimated.

### 2.5. Kinetic Characterization of rGILCC 1 and rPOXA 1B under Best Conditions Found at the Mean Comparison

**Figure 3:**
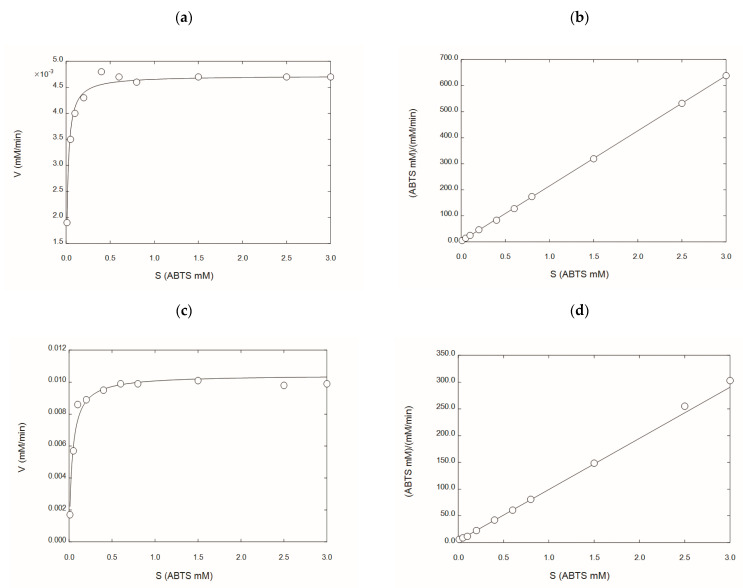
The kinetic characterization of the laccase’s enzyme concentrates in the citrate buffer rGILCC 1 and rPOXA 1B. (**a**) Michaelis–Menten plot for laccase rGILCC 1; (**b**) Hanes–Wolf linearization for laccase rGILCC 1; (**c**) Michaelis–Menten plot for laccase rPOXA 1B; (**d**) Hanes–Wolf linearization for laccase rPOXA 1B.

Figure 3 shows the Michaelis–Menten enzyme kinetics for rGILCC 1 and rPOXA 1B in the citrate buffer. Table 3 shows the kinetic parameters (*K_m_* and *V_max_*) obtained for both enzymes in citrate buffer (from this study) and acetate buffer [30,31]. The result showed that both enzymes had different Michaelis constants. The *K_m_* varied considerably between buffers. However, the citrate buffer generates higher laccase activities, which is attributed to the citrate ion, as the *K_m_* and *V_max_* of both enzymes were lower for the citrate buffer (Table 3).

### 2.6. Molecular Docking of the 3D Models of GILCC 1 and POXA 1B with ABTS at pH 3.0

**Figure 4:**
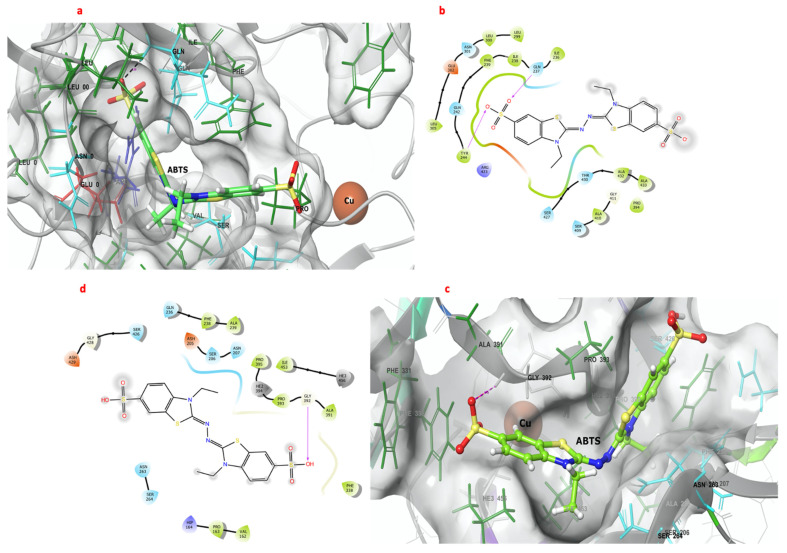
Three- and two-dimensional maps of the interactions of the 3D models of GILCC 1 and POXA 1B with ABTS at pH 3.0. (**a**) Molecular docking between the 3D model of GILCC 1 and ABTS. (**b**) Molecular interaction map of GILCC 1 and ABTS. (**c**) Molecular docking between the 3D model of POXA 1B and ABTS. (**d**) Molecular interaction of POXA 1B and ABTS.

Figure 4 shows the interactions between ABTS and the catalytic pockets of GILCC 1 and POXA 1B. The binding free energy for the complexes was −7.6 kcal mol^−1^ and −6.8 kcal mol^−1^ for GILCC 1 and POXA 1B, respectively. In the 3D and 2D contact maps between GILCC 1 and ABTS at pH 3.0 ± 0.2 (Figure 4a, b), the ligand was found to dock in the pocket and interact with the hydrophobic residues Pro^394^, Ile^236^, Ala^432^, Leu^300^, Leu^299^, Leu^305^, Ile^238^, Tyr^244^, Phe^239^, Ala^410^, and Ala^432^ and with polar residues Thr^430^, Ser^427^, Ser^409^, Asn^301^, Gln^237^, and Gln^242^. Additionally, the ligand formed hydrogen bonds with residues Tyr^244^ and Gln^237^. 

Similarly, in the case of POXA 1B and ABTS at pH 3.0 ± 0.2, the 3D and 2D contact maps (Figure 4c,d) also showed ligand binding to the pocket. Hydrophobic residues Phe^238^, Ala^239^, Pro^163^, Val^162^, Phe^330^, Ala^391^, Pro^393^, Ile^453^, and Pro^395^ as well as polar residues Ser^264^, Asn^263^, Gln^236^, Asn^207^, Ser^206^, and Ser^426^ participated in the interactions. However, in the POXA 1B–ABTS complex, hydrogen bonding was only observed with residue Gly^392^.

### 2.7. Molecular Dynamics of 3D Models of GILCC 1 and POXA 1B the Complexes GILCC 1–ABTS and POXA 1B–ABTS at pH 3.0

Both 3D models of GILCC 1 and POXA 1B [32] exhibited RMSD values of less than 2 Å throughout the trajectory (200 ns) (Supplementary Material Figure S1a). No bond breaks occurred in the active centers of the enzymes. The RMSD values indicate that the 3D structures of GILCC 1 and POXA 1B remained relatively constant during the 200 ns trajectory, with values below 2 Å. 

**Figure 5:**
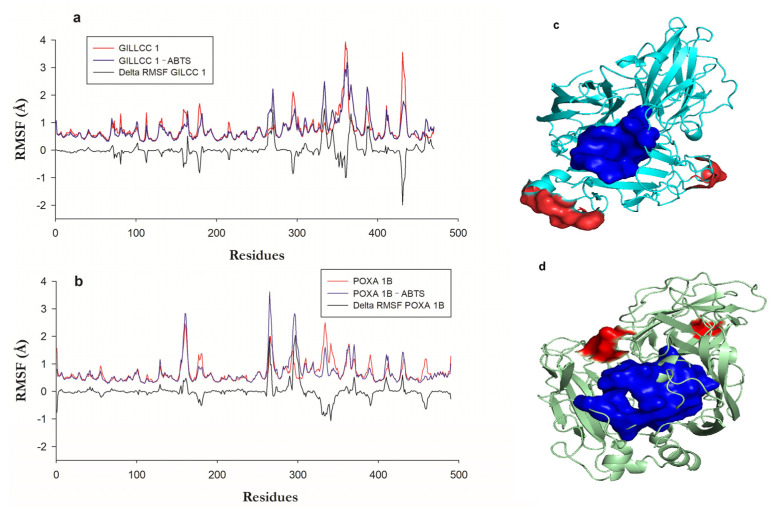
(**a**) Root means square fluctuation (RMSF) of the enzyme GILCC 1, GILCC 1–ABTS, and ΔRMSF. (**b**) Root means square fluctuation (RMSF) of the enzyme POXA 1B, POXA 1B–ABTS, and ΔRMSF POXA 1B. (**c**) Fluctuation zones of the 3D models of the enzyme GILCC 1–ABTS. (**d**) Fluctuation zones of the 3D models of the enzyme POXA 1B–ABTS.

Figure 5a show the fluctuation regions or residues of GILCC 1 and GILCC 1–ABTS along the trajectory (200 ns). In molecular dynamic simulations of GILCC 1–ABTS, ABTS remained bound to the enzyme pocket of GILCC 1 throughout the trajectory, suggesting a stable interaction inside the pocket. Amino acids such as Gln^360^, Ala^362^, Pro^431^, and Ala^432^, along with nearby residues, exhibited regions with fluctuations higher than 2 Å in the GILCC 1–ABTS complex, indicating the mobility of these amino acids and a great surrounding region. From the fluctuations, delta RMSF (∆ RMSF) calculation allows for determining the contribution of the regions and or residues in the formation of the GILCC 1–ABTS. Figure 5a shows in the profile of GILCC 1–ABTS nine negative peaks with values below −0.5 Å, showing that these regions decrease in the fluctuation due to the complex formation. The two regions with the lowest values were GLN^360^–ALA^361^ and THR^430^–GLY^434^. The GILCC 1–ABTS complex showed six fluctuating regions with more than 0.5 Å, where GLY^269^–THR^271^, ASP^331^–SER^335^, and LEU^366^–SER^370^ had the major ones. The above suggests that the ligand interacted with the regions, reducing or increasing their flexibility. The fluctuation zone LEU^366^–SER^370^ of the GILCC 1–ABTS complex is lower than that of GILCC 1. In addition, reduced fluctuation regions because of the complex formation are predominate, indicating the formation of favorable bonds and interactions. Both regions GLY^269^–THR^271^ and ASP^331^–SER^33^5 with >2 Å fluctuations do not affect the enzyme mobility because the residues involved are far from the enzyme pocket.

The end positions of the GILCC 1–ABTS complex were analyzed in the last frame of the molecular dynamics run (Supplementary Material Figure S2), proving that ABTS remained in the pocket, as the 4Å cut-off point shows relevant residues within the enzyme pocket despite the changes in enzyme and ligand configuration (Supplementary Material Video S1 and S2).

Figure 5a show the fluctuation regions or residues of POXA 1B [33] and POXA 1B–ABTS along the trajectory (200 ns). Comparison between the RMSF of POXA 1B and the RMSF of POXA 1B–ABTS shows that with the formation of the complex, the distance of the fluctuations increased. Also, ABTS detached from POXA 1B after 100 ns of simulation; before, the binding remained stable. In the interaction of the POXA 1B–ABTS complex, the protein regions with fluctuations higher than 2 Å were Leu^159^–Val^162^, Ser^264^–Gly^265^, and Asn^294^–Asn^297^, with the latter region showing the largest fluctuation in contrast to the other two fluctuating regions (Figure 5b). 

Residues like VAL^162^, ASN^263^, and Ser^264^, which interacted with ABTS and exhibited high fluctuations, were only a short distance from the ligand (2.71, 3.04, and 3.03 Å, respectively). This interaction of highly fluctuating residues with ABTS may destabilize the complex, potentially causing ABTS to exit the binding pocket, as in current research. The delta RMSF (∆ RMSF) calculation in the POXA 1B–ABTS complex showed four regions had negative ∆ RMSF, while region ALA^330^–ASP^341^ showed the lowest one (<−0.5 Å), while in the other, the fluctuations values were very close to −0.5 Å, indicating that the protein had become rigid. Five regions had the highest fluctuations, of which two showed values higher than 0.5 Å, corresponding to residues ASN^263^–PRO^267^ and ALA^295^–GLU^301^. 

The ALA^295^–GLU^301^ region is a fluctuating region associated with the formation of the complex, generating a change in the conformation of the protein backbone. Figure 5c,d show the location of the most fluctuating regions in red and the enzyme pocket residues in blue, visually identifying that in GILCC 1, the major fluctuating zones are far from the pocket (Figure 5c), while in POXA 1B, one of the fluctuating regions is closer to the enzyme pocket, affecting the interaction with the ligand during the molecular dynamics (Supplementary Material Video S3 and S4).

The resulting value of the Gibbs free energy (ΔG) calculation (using the MM-GBSA method) for the GILCC 1–ABTS complex was −20.42 kcal mol^−1^. However, the POXA 1B–ABTS complex showed instability, as ABTS detached from the POXA 1B pocket during the simulation, making the MM-GBSA calculation impossible. 

## 3. Discussion

The buffers utilized in this study are commonly reported for laccase activity measurement [34,35,36] and play a crucial role in enzyme activity. However, the enzyme activity can be affected due to biochemical reactions and enzyme interaction [37]. Because buffer solutions do not only control pH but also impact protein stability, they influence the conformation and the interface behavior [38]. Consequently, the enzyme activity is influenced by biochemical reactions and enzyme interactions, making it essential to carefully consider the choice of buffer for accurate measurements and reliable results. The statistical analysis in this study revealed the most appropriate conditions (buffers, substrate concentration, and wavelength for measurement) for rGILCC 1 and rPOXA 1B laccases activity quantification.

### 3.1. Optimal Experimental Designs of rGILCC 1 and rPOXA 1B in Acetate Buffer

The F-values of 51.40 and 202.14 for rGILCC 1 and rPOXA 1B, respectively, indicate that the models are highly significant, with only 0.01% of such large F-value occurring due to noise. A “Prob > F” value of less than 0.0500 indicates that model terms are significant. The lack-of-fit F-value for rGILCC 1 of 7.44 suggests that there was a 6.29% chance that this value could occur due to noise, making it relatively insignificant. The lack-of-fit value for rPOXA 1B is 0.09, implying that there is no significant relative to the pure error, with a 99.78% chance that it could occur due to noise. The Pred. R-squared values of 0.8224 for rGILCC 1 and 0.9774 for rPOXA 1B are in reasonable agreement with the Adj. R-squared values of 0.9489 and 0.9867, respectively, since the difference between Pred. R-squared and Adj. R-squared was less than 0.2 in both enzymes. Adequate precision (Adeq precision) measures the signal-to-noise ratio, and a ratio greater than four is considered desirable. The adequate precision ratio of 18.983 for rGILCC 1 and 39.593 for rPOXA 1B indicates that these models have navigable design spaces (Table 1), ensuring their reliability and usefulness for analysis. For rGILCC 1, the significant model terms are A-pH, B-wavelength, C-ABTS, AB, and AC, while for rPOXA 1B, they are A-pH, B-wavelength, C-ABTS, AB, AC, and BC, as shown in Table 1.

For both enzymes, the behavior in the acetate buffer was very similar. The pH had a significant (negative) effect with a contribution of 46.11% for rGILCC 1 and 53% for rPOXA 1B (Table 1), indicating that low pH values could improve the detection of the activity; therefore, consideration should be given to the laccase activity measurement at lower pH values than those used.

Wavelength also had a significant (negative) effect, with contributions of about 22.83, and 19.83% for rGILCC 1 and rPOXA 1B, respectively, indicating that low wavelength values could improve the detection of the activity. This observation shows that 420 nm was more sensitive than the 436 nm readout for detecting enzymatic reactions. Lower wavelength assays were excluded from this study since the determination of enzyme activity depended on factors like the chemical nature of the substrate, oxidation states, and molar extinction coefficient. Supporting these findings, Itoh et al. (2016) also evaluated the enzyme activity at wavelengths 420 and 436 nm for recombinant laccase Lcc2 from *Hericium coralloides* NBRC 7716. They similarly found that the 420 nm wavelength detected the oxidation of ABTS more effectively [39], aligning with our results. The choice of wavelength for a substrate transformation should technically correspond to its maximum absorption peak. For ABTS, previous studies by Wolfenden and Willson (1982) and Childs and Bardsley (1975) reported that oxidation generates an absorption peak maximum at 420 nm [40,41]. Utilizing the wavelength of the maximum absorption peak would increase sensitivity and accuracy in measuring enzyme activity.

Some researchers have used the 436 nm wavelength in their studies [42,43,44]; however, there are no reports confirming a high absorption peak at 436 nm for the oxidation of the ABTS+ radical. Instead, this wavelength is in an area of the negative slope after the point of maximum absorption. As a result, using absorbance at 436 nm could lead to an underestimation of the enzyme activity, and 415 or 420 nm wavelengths are the best and most appropriate [41,45].

The ABTS concentration showed a significant (positive) effect on enzyme activity, contributing 20.54 and 20. 73% to rGILCC 1 and rPOXA 1B, respectively. According to the results, higher substrate concentrations (>2 mM ABTS) enhance the detection of enzyme activity, which is consistent in enzymatic reactions where the enzyme active center must be saturated by the substrate for maximum reaction rate [46]. However, substrate concentrations should not be excessively high concentrations (>2 mM ABTS), as they can inhibit laccase activity [47]. Considering the kinetics, a concentration of 2 mM is adequate to ensure accurate measurements of laccase activity without unnecessary inhibition, and previous research suggests that the substrate concentration is in the excess limit [30,48].

The decisions regarding the pH conditions for the enzyme assays were due to statistical analysis. For both enzymes, the factors BC and ABC were not significant in the model (*p* > 0.05), but interactions between AB and AC were statistically significant (*p* < 0.05), with the common factor being A. In this sense, we decided to test a pH lower than the one used in the optimal experimental design (OED) (4.0 ± 0.2) (Table 2). This trial with rGILCC 1 utilized the same conditions as the best results in the ABG T2 treatment, both for the observed enzyme activity (6.30 UL^−1^) and the predicted one (6.31 UL^−1^) (Table 2). However, the pH was lowered to 3.6 ± 0.2 in the phosphate buffer (Table 2). Similarly, for the rPOXA 1B assay, the same conditions of the best result in the ABP T2 treatment were employed for both the observed (4773.66 UL^−1^) and model-predicted (4794.42 UL^−1^) enzyme activity (Table 2), also decreasing the pH to 3.6 ± 0.2 in phosphate buffer (Table 2).

Usually, enzyme activity measurement with acetate buffer is carried out at pH 4.5 ± 0.2 due to its acid dissociation constant (pKa) of 4.75 ± 0.2, leading some researchers to use acetate buffer for measuring laccase activity [35,39,44,49]. Nevertheless, the buffer pKa value allows its utilization within a pH range of 3.8 to 5.8 ± 0.2, making it suitable for use at other pH values. However, the results from this study demonstrate that for both enzymes, using a 600 mM acetate buffer at pH 4.0 ± 0.2 could improve the detection of enzyme activity, which is a buffer pH that aligns with that used by other authors to measure laccase activities [50,51].

### 3.2. Optimal Experimental Designs of rGILCC 1 and rPOXA 1B in Citrate Buffer

The F-values of 100.44 and 432.14 for rGILCC 1 and rPOXA 1B, respectively, indicate that the models are significant, and there is only a 0.01% chance that the F-value could occur due to noise. The lack-of-fit F-value of 1.18 and 4.28 implies that they were not significant relative to the pure error, suggesting 49.44 and 12.97% chances that lack-of-fit F-values could occur due to noise for rGILCC 1 and rPOXA 1B, respectively (Table 1). 

For rGILCC 1, the Pred. R-squared was 0.9686, showing reasonable agreement with the Adj. R-squared of 0.9831 because the difference between them is not higher than 0.2. The adequate precision ratio of 38.2892 indicates that the model can navigate the design space (Table 1). In this case, the factors A-pH, B-wavelength, C-ABTS, AB, and AC were significant model terms. However, as it was an irregular optimal experimental design (OED), it is not possible to estimate the effect (positive or negative) in the contribution percentage (Table 1). The pH factor was involved in both significant interactions between AB and AC, while the interactions between BC and ABC were not significant (*p* > 0.05). 

For rPOXA 1B, the Pred. R-squared of 0.9879 is reasonably in agreement with the Adj. R-squared of 0.9954 because the difference between them does not reach 0.2. The adequate precision ratio of 60.710 also indicates that this model can serve to navigate the design space (Table 1). In this case, the factors A-pH, B-wavelength, C-ABTS, AB, AC, and ABC were all significant model terms, showing that pH (a common factor in significative (*p* < 0.05) interactions: AB, AC, and ABC) must be decreased, which is supported by the fact that BC interaction was not significant (*p* > 0.05) (Table 1). 

In citrate buffer, the determination of laccase activity was higher at low pH (3.0 ± 0.2) in CBG T2 and CBP T2 treatments (Table 1 and Table 2); the results were consistent with conditions used in another research [34,36,48]. On the other hand, as pH increased (pH > 3.0 ± 0.2), the enzyme activity decreased, likely due to the pH moving away from the buffer dissociation constants (pKa), which affects the interaction of the citrate ion with the active site [52].

pH modifies the electrostatic interactions between the charged functional groups of amino acids, leading to changes in the 3D structure of proteins. Since the function of proteins depends on their specific structure, pH changes can affect the protein’s functions [52]. For example, Eichlerová et al. (2012) observed maximum enzymatic activity of laccase from *Trametes versicolor* and *Pyricularia* sp. using ABTS (as substrate) at pH of 3.0 and 4.0 ± 0.2. At basic pH, the enzyme was inhibited by hydroxyl ions, demonstrating the sensitivity of laccase activity to pH changes [53]. Notably, critical amino acids ionization, such as Asp and Gln, can occur at low and high pH levels, contributing to the pH-dependent modulation of enzyme activity [54]. 

Beyond the direct influence on enzyme activity, buffers can interact with amino acids in the active site, affecting enzyme catalysis [55]. Citrate buffer is not an exception, as citrate can form complexes with different cations, including coppers of the active center of laccases [52]. The increase in citrate concentration can decrease the reaction rate due to the presence of divalent and trivalent citrate ions, which increase their self-number with pH [56] and form more stable complexes with stronger bonds. These complexes start forming at pH 2.5 (H3CiCu), and the most stable complex occurs at pH 6.0 (CiCu^−2^), where all the cupric ions are bound to the complex [57,58]. Consequently, this reduces the availability of copper for substrate oxidation, impacting enzyme activity. In conclusion, when using citrate buffer at a pH higher than 3.0 ± 0.2, the concentration and strength of chelating citrate ions changing the coordination of copper with the conserved His residues of the active center can be observed.

Despite the apparent negative effect of citrate ions, laccases activity measurements have been possible at pH values higher than 3.0 ± 0.2 in various studies. For example, Garg et al. (2012) measured laccase activity using citrate buffer at pH 4.0 ± 0.2 [59], and Li et al. (2014) used citrate buffer at pH 4.5 ± 0.2, as it conferred higher stability to the recombinant laccase from *Trametes versicolor* [60,61]. Velásquez-Quintero et al. (2022) also investigated the laccase activity of *Pleurotus ostreatus* using different buffers, including citric acid buffer at pH 4.0, 5.0, and 6.0, and found that enzyme activity increased when the pH was more acidic [62]. However, their results differ from those obtained in the present research, where laccase activity was higher-quantified using sodium acetate buffer at pH 4.0 (50 mM) instead of sodium citrate. For rGILCC 1 and rPOXA 1B, the quantification of laccase activity at pH 4.0 ± 0.2 using acetate buffer was higher than the obtained for citrate buffer, but it did not exceed the quantification obtained at pH 3.0 ± 0.2. 

### 3.3. Low-pH Assay for rGILCC 1 and rPOXA 1B in Acetate and Citrate-Phosphate Buffers

Table 2 shows that both enzymes, rGILCC 1 and rPOXA 1B, exhibit higher enzyme activity when using the citrate-phosphate buffer (C-PBG OF and C-PBP OF) generates higher enzyme activity (7.037 ± 0.32 and 4884.26 ± 48.78 UL^−1^, respectively). However, these results do not exceed the results (7.31 ± 0.13 and 5291.67 ± 45.83 UL^−1^, respectively) obtained in the optimal experimental design (OED) of rGILCC 1 ((CBG T2) and rPOXA 1B (CBP T2), respectively), (Table 2). 

The results confirm that while pH is crucial for laccase activity quantification, the composition and ions present in the buffers can form complexes with essential ions for the enzyme’s function, potentially impacting the determination of enzyme activity [23]. The decrease in pH in citrate-phosphate buffer (C-PB) showed differences that could be due to the difference in conjugate base composition between CB and C-PB; phosphate ions can form complexes with essential ions for enzymes, which could affect the determination of enzyme activity. Cross and Cao (1999) mentioned that differently charged acid–base conjugates will be subject to differential salt effects and changes in ionization, which could vary the citrate ion charge and influence the conformation influencing laccase activity [63].

A comparison of means (Table 2) confirmed the findings obtained from the optimal experimental designs (OED) and the low-pH assay. The best conditions occurred in treatments CBG T2 for rGILCC 1 and CBP T2 for rPOXA 1B; this considerably surpasses the results obtained with the Tínoco et al. (2001) technique, as demonstrated in the present study by treatments ABG T7 for rGILCC 1 and ABP T7 for rPOXA 1B.

### 3.4. Kinetic Characterization of rGILCC 1 and rPOXA 1B under Best Conditions Found at the Mean Comparison

The generally higher *K_m_* of organic oxidases are consistent with their low substrate specificity, reflecting the ability of the enzyme–substrate binding sites to interact with different substrates. In contrast, metal oxidases typically exhibit slower turnover numbers related to organic oxidases [64].

The kinetic behavior of rGILCC 1 and rPOXA 1B, under the best conditions (Figure 2), was related to the classical enzyme activity assay frequently used in the laboratory [42]. The analysis confirmed a change in the enzyme affinity for the substrate through *K_m_* and *V_max_* using acetate and citrate buffer. For rGILCC 1, the *K_m_* went from 0.0536 to 0.00472 mM, and the *V_max_* value changed from 0.0000687 to 0.00472 mM min^−1^. Similarly, for POXA 1B, the *K_m_* value shifted from 1.716 to 0.0372 mM, and the *V_max_* value changed from 0.0316 to 0.0105 mM min^−1^.

Previously, the Michaelis–Menten apparent constant (*K_m_*) of rGILCC 1 and rPOXA 1B laccases using ABTS was determined. For rGILCC 1 a *K_m_* value of 0.0536 mM in 0.6M sodium acetate buffer at pH 4.5 [30] and for rPOXA 1B a *K_m_* value of 1.716 mM was obtained in the same buffer conditions [31]. For GILLCC 1, in the study by Sun et al. (2012), they found *K_m_* values of 0.9665 mM using 0.2 M citrate-phosphate buffer at pH 2.6 [65], while rPOXA 1B Giardina et al. (1999), obtained a *K_m_* value of 0.37 mM using McIlvaine’s citrate-phosphate buffer at pH 3.0 [66]. These findings indicate that the affinity of both enzymes changes when measured in citrate buffer.

### 3.5. GILCC 1 and POXA 1B Laccases—ABTS Molecular Docking and Molecular Dynamic

AutoDock Vina is a rapid and accurate tool for protein–ligand docking that is used to measure the binding affinity of small molecules and predict the binding poses of large substrates into catalytic protein pockets [67]. The binding affinity is determined by the stability of the enzyme–ligand complex [68], and a negative ΔG value indicates a favorable interaction, potentially leading to substrate suggesting a possible spontaneous degradation or oxidation [69]. 

In a previous study by Rivera-Hoyos et al. (2015), the molecular docking analysis between ABTS and POXA 1B resulted in a ΔG value of −4.15 kcal mol^−1^ [70], which was higher than the value obtained in this research. The discrepancy in the ΔG value could be due to the differences in the molecular docking methods used and the parameterization of the active site at pH 3.0, which may have improved affinity between ABTS and POXA 1B.

The interaction between the enzyme and the ligand depends on the type and number of residues interacting and varies depending on the substrate [71]. The conformation and interactions of ABTS with GILCC 1 and POXA 1B were similar to previous investigations. ABTS adopts a U-shaped conformation in the CuT1 pocket for both enzymes (Figure 2a,c), similar to the findings of Enguita et al. (2004), where a section of ABTS was located close to the His^497^ of copper T1 (deepest section) [72]. In the GILCC 1–ABTS complex, residues Tyr^244^ and Gly^237^ were part of the deepest section, forming hydrogen bonds with a sulphonate oxygen atom at distances of 2.07 and 2.38 Å, respectively (Figure 2a,b). Similarly, in POXA 1B, the ABTS thiazoline rings were positioned in the deepest section of the enzyme pocket, forming a hydrogen bond between a sulphonate oxygen atom and Gly^392^. These interactions in GILCC 1 are crucial for stabilizing the enzyme–substrate complex and facilitating the enzymatic reaction.

Indeed, hydrogen bonds are critical for the formation and stability of the enzyme–ligand complex, facilitating the interactions and promoting substrate catalysis, which leads to substrate degradation or oxidation [68]. However, Patil et al. (2010) pointed out that hydrophobic interactions also play a significant role in increasing substrate affinity and enhancing the biological activity of the enzyme [68]. These hydrophobic interactions contribute to stabilizing the overall biochemical environment of the complex, further enhancing its stability and function [68,69].

In GILCC 1 and POXA 1B, the residues close to CuT1 are predominantly hydrophobic, which prevents water molecules from interacting with the copper ion. This hydrophobic environment facilitates the interaction between the hydrophobic substrate and the enzyme active center [73]. The combination of hydrogen bonds and hydrophobic interactions ensures the proper orientation and positioning of the substrate within the enzyme actives centers, thus enabling effective enzymatic activity and substrate transformation.

Polar amino acids within the active sites of laccases also interact with water and have a basic side chain that accepts protons, which are crucial for substrate oxidation [74]. Some specific residues in the enzyme catalytic center identify (recognition) and accommodate the aromatic substrates with hydroxyl and amino groups. However, the identities of these interacting residues may vary depending on the substrate and the specific laccase enzyme, highlighting the importance of studying multiple substrates to understand the role of different residues in substrate recognition [75]. For example, in *T. versicolor* laccase (TvL), the Asp^206^ is a crucial residue in recognizing aromatic substrates with hydroxyl or amino groups due to its short side chain, which facilitates optimal hydrogen bond formation. However, it is remarkable that the role of Asp is pH-dependent, as its protonation state can affect its ability to accept hydrogen bonds and influence enzymatic activity accordingly [76].

The molecular dynamics analysis confirmed that the interactions and the pocket topology of GILCC 1 and POXA 1B at pH 3.0 exhibited specific behavior. Over the entire simulation, the 3D structures of both enzymes maintained a relatively constant profile (Figure 4a), as indicated by RMSD values within a window below 2 Å that remained stable during the simulations (Figure 4a). The root means square deviation (RMSD) analysis indicates that the protein skeleton maintained a constant profile, suggesting a stable conformation state [77].

However, root means square fluctuation (RMSF) analysis and ligand pocket permanence revealed distinct behaviors between GILCC 1–ABTS and POXA 1B–ABTS complexes. In the GILCC 1–ABTS complex, certain regions exhibited fluctuations similar to those in the ligand-free enzyme, but complex formation resulted in fluctuation decreasing in some areas (Figure 5). This overall fluctuation level did not compromise the stability of the GILCC 1–ABTS complex, and the presence of ABTS, even in regions away from the binding pocket, could potentially facilitate electron transfer. Conversely, in the POXA 1B–ABTS complex, fluctuating residues in the ligand-free enzyme showed increased fluctuation distances upon complex formation. Key residues such as VAL^162^ ASN^263^ and SER^264^, which interact with ABTS, displayed high fluctuations and were located at a short distance from the ligand (2.71, 3.04, and 3.03 Å, respectively). This interaction of highly fluctuating residues and ABTS could lead to complex instability, potentially causing ABTS to be pulled out of the binding pocket, as observed in the MD simulation. This phenomenon aligns with previous studies suggesting that protein structures can undergo significant rearrangement during the initial stages of molecular dynamics simulation, resulting in the loss of original contacts [78]. In this case, up to 80% of the initial contacts were lost during the first 5 ns of production due to the initial rearrangement of residues during the heating phases of the simulation and the relatively tight definition of intermolecular contacts (5 Å) [78].

Longer MD simulations provide a more accurate representation of the stability of the ligand-binding pose and its interactions with the enzyme. As the simulation progresses, the ligand can settle into a more stable binding conformation, best reflecting the actual enzyme catalysis. However, due to the slow time scale of the ligand binding and interconversion between different binding poses, ligands may get trapped in meta-stable positions during the simulations [79]. This trapping of the ligand in less stable poses can result in the loss of the more stable binding conformation and may eventually lead to the ligand exiting from the enzyme pocket altogether.

Enzyme–substrate interaction can induce change in the dynamics of enzyme active site conformational fluctuation and conformational flexibility [80]. Conformational fluctuations are crucial for substrate binding and product release; however, they may limit the rate of enzymatic reactions, as higher fluctuation of active site residues may favor higher enzymatic activity [81]. However, other authors mentioned that rigidity may also be associated with interactions generated by the surrounding residues [82], so flexible regions may be due to the loss of interactions or bonds that maintain the rigidity of the structure [32,83]. In both enzymes evaluated, the interaction with the ligand (ABTS) generated behavioral changes, as has been observed by other authors [84]. The residues with the highest fluctuation during the interaction with the ligand suggest crucial functions of these residues during the formation of the complex.

MM-GBSA values are important for validating the accuracy of compound classification based on their experimental activities and predicting the binding energy of enzyme–substrate interactions. Moreover, these values help identify the best model to explain the enzymatic activity in terms of energy [29,85]. Researchers have extensively utilized MM-GBSA/PBSA data to analyze the potential of biodegradation of various substrates during the catalysis of xenobiotic compounds. This approach provides valuable insights into the stability and energetics of enzyme–substrate interactions, shedding light on the enzymatic mechanisms underlying biodegradation processes [29,75,85]. For the GILCC 1–ABTS complex, the MM-GBSA value incorporates the dynamical receptor–ligand interaction and shows a more favorable interaction than the static interaction reported by the docking analysis; however, this dynamical interaction is opposite for the case of POXA 1B–ABTS, where the stability of the complex is lower in time, even though the docking analysis predicted similar results.

Post-docking molecular dynamics simulations provide insight into the stability of the interactions during substrate oxidation. However, it is crucial to remark that in an experimental environment, such as the one used in this study, enzyme–ABTS interactions are influenced by buffers ions. Accessing exact computational simulation of the experimental conditions is challenging, as the aqueous environment can impact the protein structure, local geometry, folding dynamics, surface charge distribution, and functionality. These changes in the protein may affect the molecule’s orientation and influence the formation of an enzyme complex [86]. Ultimately, the forces that drive the association between protein and ligands result from various interactions and energy exchanges between protein, ligand, water, and buffer ions [26]. 

## 4. Materials and Methods

### 4.1. Concentrates of Recombinant Laccases

Recombinant laccase concentrates came from individual cultures of two strains: *Pichia pastoris*/*pGAPZαA-*LacGluc-Stop (Clone 1) containing the *GILCC 1* gene from *Ganoderma lucidum* and *Pichia pastoris/pGAPZαA*-LaccPost-Stop (Clone 1) containing *POXA 1B* gene encoding Lacc6 laccase from *Pleurotus ostreatus* [44,70]. The production of each enzyme concentrate followed a previously described procedure [31]. 

### 4.2. Optimal Experimental Designs 

Two different experimental designs were employed to assess the influence of study factors on the activity of both enzymes. For the 0.6 M acetate buffer (sodium acetate and acetic acid, pH range 3.6–5.6) (AB) treatments, an optimal design (2^3^) allowed testing of two levels for each of the three factors assayed (Table 2). For the 0.1 M sodium citrate buffer (sodium citrate trihydrate and citric acid, pH range 3.0–6.2) (CB) treatments, an irregular optimal design was utilized, which included mixed levels of the three factors (Table 2). Both enzymes’ concentrates in different buffer solutions, i.e., rGILCC 1 (ABG or CBG) and rPOXA 1B (ABP or CBP), were used in the experiments. The response variable analyzed was enzyme activity (UL^−1^). The effects of factors calculations in the optimal designs were performed by an ANOVA test using Design Expert software (V 9.0).

### 4.3. Low-pH Assay (One-Factor Experimental Designs, OFED) for rGILCC 1 and rPOXA 1B Assayed in Acetate and Citrate-Phosphate

The negative effect and low contribution percentage of the pH factor evaluated during the optimal experimental designs (Table 1) indicated the necessity to assay the lowest possible pH in each of the buffers so as to determine the influence of representative ions at each buffer (citrate or acetate). The low-pH assay objective was to identify whether the pH was responsible for the increase in enzyme activity or whether the increase was due to citrate ions. To achieve a pH below pH 3.0, the citrate buffer replacement by citrate-phosphate was crucial. In the low-pH assay, both acetate buffer (AB) and citrate-phosphate buffer (citric acid and sodium hydrogen phosphate, pH range 2.6–7.0) (C-PB) were utilized with a concentration of 0.15 M (dibasic sodium phosphate dihydrate and citric acid) to evaluate rGILCC 1 (ABG OFED and C-PBG OFED). For evaluating rPOXA 1B, the buffers were ABP OFED and C-PBP OFED (Table 2). In each treatment evaluation, 2 mM of ABTS was the substrate, and measurement of the absorbance occurred at a wavelength of 420 nm.

### 4.4. Assay for rGILCC1 and rPOXA 1B in Britton–Robinson Buffer at pH 3.0 and 4.0 ± 0.2

To determine the influence the buffer ions had on the two pH values used in other investigations (citrate buffer pH 3.0 ± 0.2 and acetate buffer pH 4 ± 0.2), the use of Britton–Robinson buffer, which is a universal pH buffer, allowed a pH range from 2 to 12. Laccase activity was evaluated at pH 3.0 and 4 ± 0.2 in 0.04 M Britton–Robinson buffer (B-RB) (acetic acid, phosphoric acid, and boric acid) for both rGILCC 1 (B-RBG) and rPOXA 1B (B-RBP) enzymes. In the assessment of treatments, ABTS (2 mM) was the substrate, and enzyme activity quantification was carried out at a wavelength of 420 nm.

### 4.5. Laccase Activity Assay Using Acetate Buffer

The enzyme activity was monitored by measuring the change in absorbance at different wavelengths, each tested with its corresponding molar extinction coefficient resulting from the oxidation of ABTS in 600 mM sodium acetate buffer at different pH levels. The detection mixture consisted of 800 µL of crude extract at room temperature, 100 µL (600 mM) of sodium acetate buffer, and 100µL of (5 mM) of ABTS. The formation of the green cation radical was spectrophotometrically measured 3 min after the start of the enzymatic reaction. The blank solution contained 800 µL of distilled water, 100 µL (600 mM) of sodium acetate buffer, and 100 µL of ABTS (at the different substrate concentrations tested). The enzyme activity expression was in U L^−1^ [42]. 

### 4.6. Laccase Activity Assay Using Citrate Buffer

Laccase activity was determined by measuring the oxidation of ABTS as substrate, using changes in absorbance at different wavelengths with their respective molar extinction coefficient, in 0.1 M sodium citrate buffer at different pH levels. The reaction occurred at room temperature with 100 μL of 20 mM ABTS and 2 to 20 μL of centrifuged supernatant. (The volume varied depending on the enzyme concentration in the sample.) The adjusted reaction volume was 1 mL, containing 0.1 M citrate buffer (prepared at different pH levels). The formation of the green cation radical was spectrophotometrically measured 1 min after the start of the enzymatic reaction [48]. The reaction blank contained 20 µL distilled water, 900 µL (0.1 M) sodium citrate buffer, and 100 µL of ABTS (at the different substrate concentrations tested). The enzyme activity expression was in U L^−1^ [42]. 

The enzyme activity unit definition is the amount of enzyme required to oxidize 1 µmol ABTS per minute (Equation (1)).

(1) $${UL}^{-1}=\left(\frac{\frac{E}{t}}{ɛ}\right)\times \left(\frac{V_{t}}{V_{s}}\right)\times 10^{6}\times fd$$

where Δ*E* corresponds to the change in absorbance over the reaction time (final absorbance—initial absorbance). ε, the molar extinction coefficient of ABTS (M^−1^ cm^−1^), depends on the wavelength used (for 436 nm, ɛ is 29,300 M^−1^ cm^−1^; and for 420 nm, ɛ is 36,000 M^−1^ cm^−1^); *V_t_* represents the total reaction volume in mL, and *Vs* is the sample volume (mL) contained in the reaction. The conversion factor 10^6^ allows moles of ABTS transformation to micromoles, and *fd* corresponds to the dilution factor applied to the sample.

### 4.7. Mean Comparison

Mean comparison between optimal experimental design (OED) and one-factor experimental designs (OFED) treatments was possible by using Duncan’s test, which is part of the SPSS software (V 24.0) with a confidence interval of 95% (α 0.05).

### 4.8. Kinetic Characterization of rGILCC 1 and rPOXA 1B under Best Conditions Found at the Mean Comparison

The apparent kinetic constants of the concentrates of rGILCC 1 and rPOXA 1B were accessed using ABTS as the substrate in the concentration range of 0.01–3.00 mM. The experiments involved two different buffers: 100 mM of sodium citrate buffer at pH 3.0 ± 0.2 and 600 mM of acetate buffer at pH 4.5 ± 0.2. The starting enzyme solution activity set-up was 10.7 UL^−1^, and the temperature was 25 °C. The Michaelis–Menten equation served to fit the hyperbola and obtain the apparent *K_m_* and *V_max_* using SIMFIT Software (V 7.4.6) [87] following the Hanes–Woolf linear regression [46]. 

### 4.9. Three-dimensional Computational Homology and Metal Ion Modelling of GILCC 1 and POXA 1B Laccases

Due to the absence of a previously reported crystallographic structure for GILCC 1 from *Ganoderma lucidum*, a 3D model of the enzyme was built. To identify and remove amino acids from the signal peptide, the enzyme sequence was fed into the SignalP 5.0 server (http://www.cbs.dtu.dk/services/SignalP/ (accessed on 20 January 2023)). The 3D structure of the laccase was predicted and analyzed following the methodology utilized by Ardila-Leal et al. (2021) and generated using the Swiss-model server. All coppers (Cu T1 and Cu from TNC) were modelled as Cu^2+^ in the GILCC 1 binding pocket using the metal center parameter builder based in Python (MCPB.py) [32,83], which consists of employing two models to achieve a balance between precision and speed, thus obtaining parameters of bonds, angles, and partial charges of the metal ions and the residues of the active center [88], included in the Amber tools 18 package [89]. The molecule’s partial charges resulted from the electrostatic calculation using Gaussian calculations [90]. 

The generated 3D model of the GILCC 1 enzyme used in this study was validated and parameterized at pH 3.0 following the same methodology employed for the POXA 1B model [32]. 

### 4.10. GILCC 1–ABTS and POXA 1B–ABTS Molecular Docking and Dynamics

#### 4.10.1. Enzyme and Ligand Preparation

The H^++^ server (http://biophysics.cs.vt.edu/ (accessed on 20 January 2023)) was employed to determine the protonation state of the 3D models of GILCC 1 and POXA 1B side chains at pH 3.0, calculating acidity constants in a local and automated way (Supplementary Material Table S1). Protein preparation incorporated the polar hydrogens and the ff14SB force field to model all laccase residues during parametrization [39]. The crystallographic structure of the ABTS (ligand) was extracted from the 3ZDW (CotA laccase—*Bacillus subtilis*) reported in the Protein Data Bank (PDB). Ligand protonation based on pH and energy minimization was performed by using Avogadro software version 1.97. In AutoDock, the assignment of the structure twist number was automated, allowing flexible structures during the molecular docking calculation. After adding the polar hydrogens, charge assignments were carried out using the Gasteiger method.

#### 4.10.2. Molecular Docking

Protein–ligand docking analysis was carried out using AutoDock Tools and AutoDock Vina 4 software. The grid calculation used the FTsite program (https://ftsite.bu.edu/ (accessed on 20 January 2023)) [91] to identify the residues of the catalytic pocket. The grid construction, with dimensions of 26 × 26 × 30 and spacing of 0.375 Å, was focused on CuT1. Ten enzyme–ligand docking assays were conducted with a completeness of 20, using rigid receptors while keeping the ABTS flexible. Finally, choosing the lowest Gibbs energy (kcal mol^−1^) of the enzymes–ABTS docking results allowed subsequent molecular dynamics simulations.

#### 4.10.3. Molecular Dynamics

Molecular dynamics simulations were performed by using Ambertools 18 [89] for the 3D models of GILCC 1 and POXA 1B and for GILCC 1–ABTS and POXA 1B–ABTS complexes. The topology files and the system coordinates preparation were performed by tleap program use. Each enzyme–ligand complex was immersed in a TIP3P water box [92] with a dimension of 10.0 Å. 

Each system neutralization involved Na^+^ and Cl^−^ ions addition to set an ionic strength of 150 mM. Topology and coordinate files for the simulation steps were then generated. Initial energy minimization was carried out using 10,000 steps of the steepest descent algorithm steps, followed by 1000 steps of conjugate gradients. The minimized system was gradually heated up to 300 K during 200 ps using a canonical ensemble (NVT) with the Langevin thermostat set at a collision frequency of 2.0 ps^−1^ and a step size of 2 fs. Subsequently, a density simulation was performed for 1 ns, maintaining the temperature at 300 K and using an isothermal-isobaric assembly (NPT) with hydrogen bond length constraints (SHAKE). For the production step, the previous conditions remained unchanged for 200 ns. Each enzyme–ABTS complex, molecular dynamics was carried out in duplicate to validate the results.

Trajectory files were recorded every 0.2 ns used to detect changes in the enzyme–ligand complex geometry. The CPPTRAJ tool allowed knowing the trajectory data behavior, calculating the root mean square deviation (RMSD), root mean square fluctuation (RMSF), and Gibbs free energy (ΔG) using the MM-GBSA methodology [93].

## 5. Conclusions

The measurement of laccase activity depends on several factors, such as buffer choice, pH, ABTS concentration, and wavelength, for activity measurement. In this research, both rGILCC 1 and rPOXA 1B showed similar standardized conditions for laccase activity detection when utilizing citrate buffer at pH 3.0 ± 0.2, a wavelength 420 nm (ɛ = 36,000 M^−1^ cm^−1^), and an ABTS concentration of 2 mM. The enzyme kinetics experiments revealed that these standardized conditions with citrate buffer enhanced the affinity for ABTS compared to the acetate buffer. The presence of citrate ions seemed to influence enzyme activity, particularly for copper-containing metalloenzymes. 

The study further employed molecular docking of GILCC 1 and POXA 1B with ABTS, highlighting the relevance of the substrate binding pockets conformation and the residues identified within the enzyme pocket. Furthermore, molecular dynamics simulations provided valuable insights into the contacts, interactions, and fluctuation effects on the enzyme, offering an approximation of the phenomena occurring during the enzymatic catalysis. It is obvious that reproducing all the conditions or changes that affect the enzyme-substrate interaction can be challenging, both experimentally and computationally.

Overall, these findings contribute to a deeper understanding of laccase activity and its regulation, highlighting the importance of standardized conditions and the role of citrate ions in influencing enzyme behavior.

## Figures and Tables

**Table 1:** Statistical results (ANOVA) of the optimal experimental designs (OED) in acetate buffer and citrate buffer for rGILCC 1 and rPOXA 1B enzymes, effect, and percentage contribution of the factors on laccase activity (UL^−1^).

**Source**	**rGILCC 1 (Acetate Buffer)**					**rPOXA 1B (Acetate Buffer)**				
	**F**	***p*-value**	**Effect**	**%**		**F**	***p*-value**	**Effect**	**%**	
	**Value**	**Prob > F**		**Contribution**		**Value**	**Prob > F**		**Contribution**	
Model	51.40	<0.0001				202.14	<0.0001			
A-pH	166.98	<0.0001	−1.36	46.11		783.01	<0.0001	−1524.96	53.62	
B-wavelength	82.66	<0.0001	−0.96	22.83		290.63	<0.0001	−929.06	19.90	
C-ABTS	74.37	<0.0001	0.91	20.54		302.73	<0.0001	948.21	20.73	
AB	5.89	0.0320	0.26	1.63		35.71	<0.0001	325.65	2.45	
AC	15.88	0.0018	−0.42	4.39		26.96	0.0002	282.98	1.85	
BC	1.49	0.2460	0.13	0.41		7.27	0.0194	146.97	0.50	
ABC	2.85	0.1172	−0.18	0.79		1.95	0.1874	−76.19	0.13	
Lack of fit	7.44	0.0629				0.09	0.9978			
R-squared					0.9677					0.9916
Adj R-squared					0.9489					0.9867
Pred R-squared					0.8224					0.9774
Adeq precision					18.983					39.593
**Source**	**rGILCC1 (Citrate Buffer)**					**rPOXA 1B (Citrate Buffer)**				
	**F**	***p*-value**				**F**	***p*-value**			
	**Value**	**Prob > F**				**Value**	**Prob > **F			
Model	100.44	<0.0001				432.14	<0.0001			
A-pH	306.42	<0.0001				1740.36	<0.0001			
B-wavelength	351.98	<0.0001				621.23	<0.0001			
C-ABTS	74.58	<0.0001				487.05	<0.0001			
AB	20.03	0.0002				55.33	<0.0001			
AC	13.64	0.0010				44.23	<0.0001			
BC	0.03	0.8583				2.19	0.167			
ABC	0.50	0.6205				8.34	0.006			
Lack of fit	1.18	0.4944				4.28	0.130			
R-squared					0.9892					0.9977
Adj R-squared					0.9831					0.9954
Pred R-squared					0.9686					0.9879
Adeq precision					38.2892					60.7103

A, assay factor code for pH; B, assay factor code for wavelength; C, assay factor code for ABTS concentration; AB, AC, BC, and ABC, factors code combinations at the different experimental designs.

**Table 2:** Treatments and results achieved by the response variable (UL^−1^ enzyme activity) for each enzyme under the conditions tested at Optimal Experimental Designs (OED) and Low pH results for rGILLC 1 and rPOXA 1B assayed in acetate buffer (AB) and citrate-phosphate buffer (C-PB) at low-pH assay.

**Treatments**		**rGILCC 1 at Optimal Experimental Designs (OED)**						
		**Factors**			**Enz. Act. (UL^−1^)**			
		**A**	**B**	**C**	**Acetate Buffer (AB)**		**Citrate Buffer (CB)**	
**Acetate Buffer**	**Citrate Buffer**	**pH**	**Wavelength (nm)**	**ABTS (mM)**	**Observed**	**Predicted**	**Observed**	**Predicted**
	CBG T1	3.00	420	0.50			7.04 ± 0.00 ^a^	7.12 ± 0.00
	CBG T2	3.00	420	2.00			7.31 ± 0.13 ^a^	7.30 ± 0.00
	CBG T3	3.00	436	0.50			5.35 ± 0.16 ^d,e,f^	5.33 ± 0.00
	CBG T4	3.00	436	2.00			5.46 ± 0.00 ^d,e^	5.40 ± 0.00
ABG T1	CBG T5	4.00	420	0.50	5.31 ± 0.06 ^d,e,f^	5.31 ± 0.00	4.72 ± 0.13 ^g^	4.57 ± 0.00
ABG T2	CBG T6	4.00	420	2.00	6.30 ± 0.17 ^c^	6.31 ± 0.00	5.56 ± 0.00 ^d^	5.63 ± 0.00
ABG T3	CBG T7	4.00	436	0.50	3.74 ± 0.04 ^j^	3.79 ± 0.00	3.64 ± 0.32 ^j^	3.72 ± 0.00
ABG T4	CBG T8	4.00	436	2.00	5.08 ± 0.00 ^f^	5.44 ± 0.00	4.70 ± 0.35 ^g^	4.61 ± 0.00
ABG T5	CBG T9	4.50	420	0.50	3.93 ± 0.07 ^i,j^	3.93 ± 0.00	4.63 ± 0.00 ^g^	4.71 ± 0.00
ABG T6	CBG T10	4.50	420	2.00	4.47 ± 0.04 ^g,h^	4.46 ± 0.00	5.19 ± 0.00 ^e,f^	5.13 ± 0.00
ABG T7	CBG T11	4.50	436	0.50	2.81 ± 0.11 ^k^	3.30 ± 0.00	3.75 ± 0.16 ^j^	3.70 ± 0.00
ABG T8	CBG T12	4.50	436	2.00	3.69 ± 0.05 ^j^	3.70 ± 0.00	4.21 ± 0.16 ^h,i^	3.70 ± 0.00
**Treatments**		**rPOXA 1B at Optimal Experimental Designs (OED)**						
		**Factors**			**Enz. Act. (UL^−1^)**			
		**A**	**B**	**C**	**Acetate Buffer (AB)**		**Citrate Buffer (CB)**	
**Acetate Buffer**	**Citrate Buffer**	**pH**	**Wavelength (nm)**	**ABTS (mM)**	**Observed**	**Predicted**	**Observed**	**Predicted**
	CBP T1	3.00	420	0.50			4962.96 ± 13.09 ^b^	4958.89 ± 0.00
	CBP T2	3.00	420	2.00			5291.67 ± 45.83 ^a^	5291.68 ± 0.00
	CBP T3	3.00	436	0.50			2870.37 ± 130.95 ^e^	2870.39 ± 0.00
	CBP T4	3.00	436	2.00			3180.56 ± 124.40 ^d^	3184.61 ± 0.00
ABP T1	CBP T5	4.00	420	0.50	4378.60 ± 28.13 ^c^	4378.60 ± 0.00	2236.11 ± 32.74 ^i,j^	2244.25 ± 0.00
ABP T2	CBP T6	4.00	420	2.00	4773.66 ± 95.15 ^b^	4794.42 ± 0.00	3870.37 ± 13.09 ^d^	3866.30 ± 0.00
ABP T3	CBP T7	4.00	436	0.50	2889.64 ± 238.37 ^g^	2910.40 ± 0.00	1300.93 ± 72.02 ^k^	1296.86 ± 0.00
ABP T4	CBP T8	4.00	436	2.00	3804.51 ± 1.34 ^d^	3786.64 ± 0.00	2067.90 ± 95.03 ^h^	2073.32 ± 0.00
ABP T5	CBP T9	4.50	420	0.50	2160.49 ± 25.26 ^i,j^	2160.49 ± 0.00	1643.52 ± 32.74 ^k^	1639.45 ± 0.00
ABP T6	CBP T10	4.50	420	2.00	3320.47 ± 59.46 ^f^	3320.47 ± 0.00	2254.63 ± 32.74 ^i^	2258.68 ± 0.00
ABP T7	CBP T11	4.50	436	0.50	1518.77 ± 12.07 ^k^	1500.89 ± 0.00	912.04 ± 19.64 ^l^	916.09 ± 0.00
ABP T8	CBP T12	4.50	436	2.00	2773.35 ± 162.24 ^g^	2788.35 ± 0.00	1666.67 ± 52.38 ^j^	1658.56 ± 0.00
**Treatments**		**rGILCC 1 and rPOXA 1B at low-pH assay**						
		**Factor**	**Fixed terms**		**Enz. Act. (UL^−1^)**			
		**A**	**B**	**C**	**Acetate Buffer (AB OFED)**		**Citrate-Phosphate Buffer (C-PB OFED)**	
		**pH**	**Wavelength (nm)**	**ABTS (mM)**	**Observed**		**Observed**	
ABG OFED		3.6	420	2	6.67 ± 0.19 ^b^			
ABP OFED		3.6	420	2	4819.44 ± 55.56 ^b^			
	C-PBG OFED	2.6	420	2			7.037 ± 0.32 ^a^	
	C-PBP OFED	2.6	420	2			4884.26 ± 48.78 ^b^	

Differences among mean ± SD are represented by letters as established by Duncan’s test, applied on the observed data (significant differences, *p* < 0.05). Error bars correspond to SD.

**Table 3:** *K_m_* and *V_max_* kinetic parameters comparison among rGILCC 1 and rPOXA 1B enzymes in citrate and acetate buffer.

Enzyme	Buffer	Apparent Kinetic Parameters		SEM	Confidence Limit 95%		*p*	References
rGILCC 1	Citrate	*V_max_* _(mM min^−1^)_	4.72 × 10^−3^	1.67 × 10^−7^	4.72 × 10^−3^	4.72 × 10^−3^	<0.001	This study
		*K_m_* _(mM)_	1.49 × 10^−2^	2.34 × 10^−6^	1.48 × 10^−2^	1.49 × 10^−2^	<0.001	
	Acetate	*V_max_* _(mM min^−1^)_	6.87 × 10^−5^	1.16 × 10^−5^	3.92 × 10^−3^	3.99 × 10^−3^	<0.001	[30]
		*K_m_* _(mM)_	5.36 × 10^−2^	5.17 × 10^−4^	4.75 × 10^−2^	5.03 × 10^−2^	<0.001	
rPOXA 1B	Citrate	*V_max_* _(mM min^−1^)_	1.05 × 10^−2^	3.20 × 10^−4^	9.70 × 10^−3^	1.12 × 10^−2^	<0.001	This study
		*K_m_* _(mM)_	3.72 × 10^−2^	5.07 × 10^−3^	2.56 × 10^−2^	4.89 × 10^−2^	<0.001	
	Acetate	*V_max_* _(mM min_^−1^_)_	3.16 × 10^−2^	1.0 × 10^−3^	2.90 × 10^−2^	3.40 × 10^−2^	<0.001	[31]
		*K_m_* _(mM)_	1.72	1.21 × 10^−1^	1.44	1.99	<0.001	

## Data Availability

Data are contained within the article.

## Supplementary Materials

The following supporting information can be downloaded at: https://www.mdpi.com/article/10.3390/molecules28217263/s1. Figure S1. Root Mean Square Deviation (RMSD). a. RMSD values of the complexes. GILCC 1-ABTS and POXA 1B-ABTS, at pH 3, along the trajectory (200 ns). b. RMSD of ligand and receptor in dynamics assay 1. c. RMSD of ligand and receptor in dynamics assay 2. Figure S2. 2D contact maps of last frame interactions (GILCC 1-ABTS). a. First molecular dynamics assay. b. Second molecular dynamics assay. Table S1. Amino acid protonation state at pH 3.0 of GILCC1 and POXA 1B. Videos 1–4 are in https://figshare.com/account/items/24297490/edit (accesssed on 20 January 2023).

## Sample Availability

Samples of the compounds are available from the authors.
