# Supplementary material for: Statistical Improvement of rGILCC 1 and rPOXA 1B Laccases Activity Assay Conditions Supported by Molecular Dynamics
Source: Molecules. 2023 Oct 25;28(21):7263. doi: 10.3390/molecules28217263 (PMC10648076; doi:10.3390/molecules28217263)
Supplement: Supplementary file 1 [file molecules-28-07263-s001.zip › molecules-2623434-supplementary.pdf]

Supplementary Material

Statistical Improvement of rGILCC 1 and rPOXA 1B Laccases Activity Assay Conditions Supported by Molecular Dynamics

María P. C. Mora-Gamboa [1,†], María C. Ferrucho-Calle [1,†], Leidy D. Ardila-Leal [1,2], Lina M. Rojas-Ojeda [3], Johan F. Galindo [3,*], Raúl A. Poutou-Piñales [1,*], Aura M. Pedroza-Rodríguez [4] and Balkys E. Quevedo-Hidalgo [5]

[1]Laboratorio de Biotecnología Molecular, Grupo de Biotecnología Ambiental e Industrial (GBAI), Departamento de Microbiología, Facultad de Ciencias, Pontificia Universidad Javeriana, Bogotá 110231, Colombia
[2]Laboratorio de Biotecnología Vegetal, Grupo de Investigación en Asuntos Ambientales y Desarrollo Sostenible (MINDALA), Departamento de Ciencias Agrarias y del Ambiente, Universidad Francisco de Paula Santander, Ocaña 546552, Colombia
[3]Departamento de Química, Universidad Nacional de Colombia, Bogotá 111321, Colombia
[4]Laboratorio de Microbiología Ambiental y Suelos, Grupo de Biotecnología Ambiental e Industrial (GBAI), Departamento de Microbiología, Facultad de Ciencias, Pontificia Universidad Javeriana, Bogotá 110231, Colombia
[5]Laboratorio de Biotecnología Aplicada, Grupo de Biotecnología Ambiental e Industrial (GBAI), Departamento de Microbiología, Facultad de Ciencias, Pontificia Universidad Javeriana, Bogotá 110231, Colombia

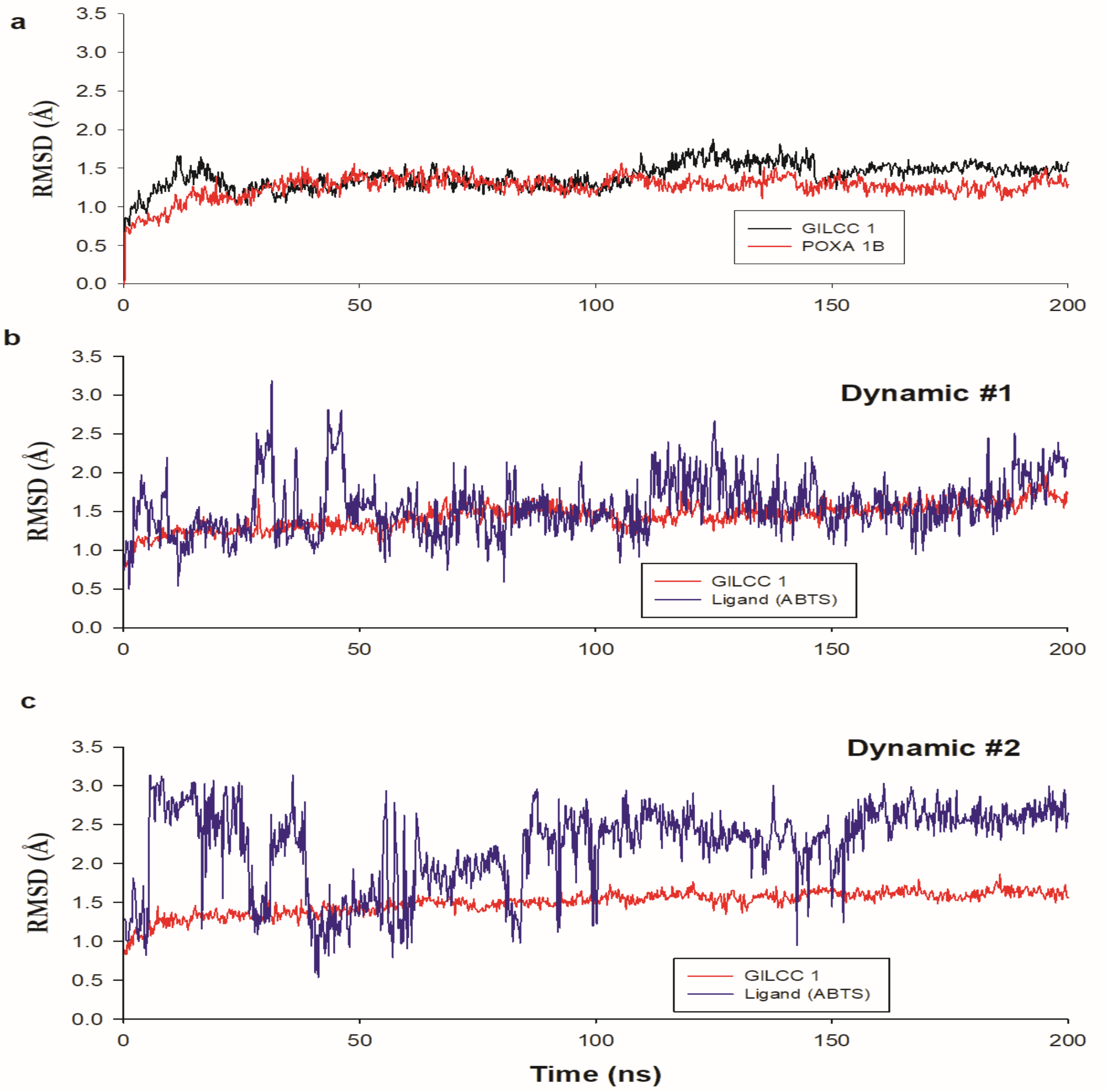

Fig. S1. **Root Mean Square Deviation (RMSD). a.** RMSD values of the complexes. GILCC 1-ABTS and POXA 1B-ABTS, at pH 3, along the trajectory (200 ns). **b.** RMSD of ligand and receptor in dynamics assay 1. c. RMSD of ligand and receptor in dynamics assay 2.

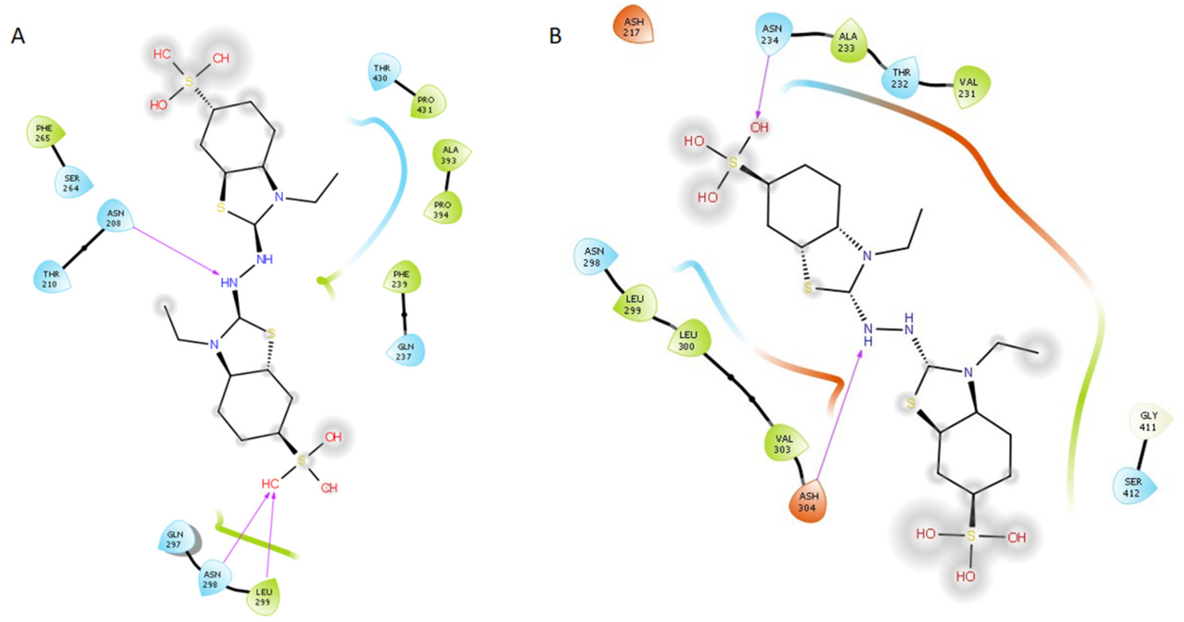

Fig. S2. **2D contact maps of last frame interactions (GILCC 1-ABTS)**. **a**. First molecular dynamics assay. **b**. Second molecular dynamics assay

Table S1. Amino acid protonation state at pH 3.0 of GILCC1 and POXA 1B

GILCC 1						POXA 1B					
Residue		pKa	Residue		pKa	Residue		pKa	Residue		pKa
ASP	18	2.84	LYS	40	10.22	ASP	18	3.14	LYS	37	10.36
ASP	96	3.07	LYS	59	10.43	ASP	42	2.55	LYS	39	10.29
ASP	101	3.8	LYS	71	10.06	ASP	77	6.95	LYS	51	10.29
ASP	118	1.37	LYS	130	10.5	ASP	96	3.49	LYS	71	10.36
ASP	128	2.75	LYS	134	10.22	ASP	118	1	LYS	129	10.5
ASP	131	0.77	LYS	194	10.5	ASP	131	1.15	LYS	134	10.36
ASP	138	2.81	LYS	311	8.95	ASP	138	2.7	LYS	191	10.43
ASP	140	-2.39	LYS	378	10.5	ASP	140	-1.91	LYS	193	10.5
ASP	150	1.74	ARG	22	12.16	ASP	150	1.11	LYS	281	10.5
ASP	167	3.19	ARG	43	12.15	ASP	166	4.2	LYS	309	10.5
ASP	214	-0.06	ARG	121	10.33	ASP	213	-0.48	LYS	310	12.03
ASP	224	-0.86	ARG	157	12.06	ASP	223	-2.14	LYS	315	10.5
ASP	323	1.91	ARG	176	11.87	ASP	233	1.69	LYS	376	10.5
ASP	424	7.29	ARG	195	11.59	ASP	322	2.63	ARG	5	12.5
ASP	470	3.88	ARG	197	18.29	ASP	362	4.01	ARG	22	11.74
GLU	222	1.7	ARG	199	13.73	ASP	365	2.1	ARG	43	12.01
GLU	302	4.1	ARG	243	11.53	ASP	423	4.15	ARG	121	11.91
GLU	381	0.76	ARG	260	13.32	ASP	430	2.59	ARG	135	12.22
GLU	469	4.44	ARG	280	11.38	ASP	442	3.93	ARG	175	11.31
CYS	85	9	ARG	316	12.5	GLU	142	4.65	ARG	194	11.66
TYR	20	10	ARG	408	11.45	GLU	221	0.67	ARG	196	17.53
TYR	95	10.54	ARG	423	14.68	GLU	225	3.78	ARG	198	14.6
TYR	108	12.5	ARG	440	13.22	GLU	301	3.19	ARG	242	12.62
TYR	116	12.86				GLU	382	1.28	ARG	259	13.38
TYR	127	11.21				GLU	467	6.28	ARG	279	11.45
TYR	137	11.38				TYR	21	10	ARG	284	12.5
TYR	152	15.52				TYR	31	11.13	ARG	305	12.5
TYR	196	9.04				TYR	95	11.63	ARG	361	12.5
TYR	209	14.15				TYR	108	12.11	ARG	407	11.1
TYR	244	11.57				TYR	116	12.63	ARG	421	11.29
TYR	257	9.45				TYR	127	12.08	ARG	422	14.77
TYR	281	9.76				TYR	137	11.12	ARG	438	10.82
TYR	372	10.87				TYR	152	14.42			
TYR	415	9.27				TYR	185	10.6			
TYR	417	10.85				TYR	195	8.98			
						TYR	208	12.11			
						TYR	243	11.47			
						TYR	256	10.14			
						TYR	280	10.24			
						TYR	302	10.07			
						TYR	373	9.66			
						TYR	414	8.75			

						TYR	416	13.01			
						TYR	489	10			
